# Probing the requirement for CD38 in retinoic acid-induced HL-60 cell differentiation with a small molecule dimerizer and genetic knockout

**DOI:** 10.1038/s41598-017-17720-4

**Published:** 2017-12-12

**Authors:** Robert J. MacDonald, Jonathan H. Shrimp, Hong Jiang, Lu Zhang, Hening Lin, Andrew Yen

**Affiliations:** 1 Department of Biomedical Sciences, Cornell University, Ithaca, NY 14853 USA; 2 Department of Chemistry and Chemical Biology, Cornell University, Ithaca, NY 14853 USA; 3 Howard Hughes Medical Institute, Department of Chemistry and Chemical Biology, Cornell University, Ithaca, NY 14853 USA

## Abstract

CD38 is an ectoenzyme and receptor with key physiological roles. It metabolizes NAD^+^ to adenosine diphosphate ribose (ADPR) and cyclic ADPR, regulating several processes including calcium signalling. CD38 is both a positive and negative prognostic indicator in leukaemia. In all-*trans* retinoic acid (RA)-induced differentiation of acute promyelocytic leukaemia and HL-60 cells, CD38 is one of the earliest and most prominently upregulated proteins known. CD38 overexpression enhances differentiation, while morpholino- and siRNA-induced knockdown diminishes it. CD38, via Src family kinases and adapters, interacts with a MAPK signalling axis that propels differentiation. Motivated by evidence suggesting the importance of CD38, we sought to determine whether it functions via dimerization. We created a linker based on the suicide substrate arabinosyl-2′-fluoro-2′-deoxy NAD^+^ (F-araNAD^+^), dimeric F-araNAD^+^, to induce homodimerization. CD38 homodimerization did not affect RA-induced differentiation. Probing the importance of CD38 further, we created HL-60 cell lines with CRISPR/Cas9-mediated CD38 truncations. Deletion of its enzymatic domain did not affect differentiation. Apart from increased RA-induced CD11b expression, ablation of all but the first six amino acids of CD38 affected neither RA-induced differentiation nor associated signalling. Although we cannot discount the importance of this peptide, our study indicates that CD38 is not necessary for RA-induced differentiation.

## Introduction

CD38 is a type II transmembrane ectoenzyme and receptor with roles in numerous physiological processes and malignancies. Through its extracellular enzymatic activity, which converts NAD^+^ to adenosine diphosphate-ribose (ADPR) and, to a small extent, cyclic ADPR, CD38 regulates calcium signalling and thus affects many processes such as bone metabolism, insulin secretion, and oxytocin secretion^1–5^. CD38 also acts as a receptor and is involved in B and T lymphocyte activation, as well as leukocyte adhesion^6–8^. It is a negative prognostic indicator in HIV and chronic lymphocytic leukaemia, but a positive prognostic indicator in acute myeloid leukaemia^9–12^. Its attributes as a receptor giving rise to these different effects are ergo of interest to both pathogenesis and therapy.

Several pieces of evidence suggest that CD38 may be a key driver of all-*trans* retinoic acid (RA)-induced differentiation. In HL-60 acute myeloid leukaemia cells, a model for RA-induced differentiation, RA induces dramatic upregulation of CD38 mRNA and protein expression within 8 h^13–16^. Overexpression of CD38 enhances RA-induced myelo-monocytic differentiation evidenced by markers including CD11b expression, G_1_/G_0_ cell cycle arrest, and inducible reactive oxygen species production^17^. Conversely, morpholino- and siRNA-mediated knockdown of CD38 cripples RA-induced differentiation^18^. CD38 interacts with a number of receptor-regulated/associated signalling molecules including c-Cbl, Slp76, Vav1, and Lyn, several adapters and a Src family kinase^19–21^. These proteins also interact with one another^19–21^. Further, Lyn is linked to c-Raf and ERK, members of a MAPK signalling cascade necessary for RA-induced differentiation^22–24^. Ligation of CD38 using its ligand, CD31, or anti-CD38 monoclonal antibodies induces activation of a multitude of signalling proteins, including c-Cbl, p85 PI3K, c-Raf, and ERK, as well as secretion of cytokines such as IL-6 and IL-10^8,20,25–29^.

These clues drove us to investigate what CD38 receptor functions might contribute to RA-induced differentiation. We considered three classical receptor signal transduction mechanisms by which CD38 might act. One is via signalling dependent on its intracellular domain. The cytosolic tail, however, is very short, comprising only 21 amino acids. Another is through its extracellular domain, which possesses its catalytic activity. A previous report discounted this, demonstrating that CD38 catalytic activity is not necessary for RA-induced differentiation^30^. Finally, CD38 might propel RA-induced differentiation through dimerization-induced signalling, which might depend on extracellular and intracellular domains and be induced by its ligand, CD31, or by CD38 antibodies. We viewed dimerization as particularly attractive since it is not only one of the most common mechanisms by which transmembrane receptors function, but it could also explain the ensemble of interactions between CD38 and differentiation-driving cytosolic signalling proteins. Since CD38 has a short cytosolic tail, dimerization would allow for a larger area upon which these interactions could occur. A prior study has also found that, in murine B lymphocytes, CD38 is expressed as non-covalently associated homodimers^31^. We thus opted to investigate dimerization as a potential differentiation-driving signalling modality of CD38.

To assess the effects of CD38 dimerization on RA-induced differentiation, we used a small molecule dimerizer of CD38 to induce CD38 homodimer formation. We found that CD38 dimerization did not affect RA-induced differentiation in HL-60 cells. To further probe the role of CD38 in the process, we then used the CRISPR/Cas9 system to disrupt the CD38 gene in HL-60 cells. Surprisingly, we found that apart from an increase in CD11b in one of three lines, ablation of all but six amino acids of the wild-type protein had no effect on RA-induced differentiation, suggesting that the CD38 is not necessary for the process to occur.

## Results

### Design and synthesis of a small molecule dimerizer of CD38

**Figure 1:**
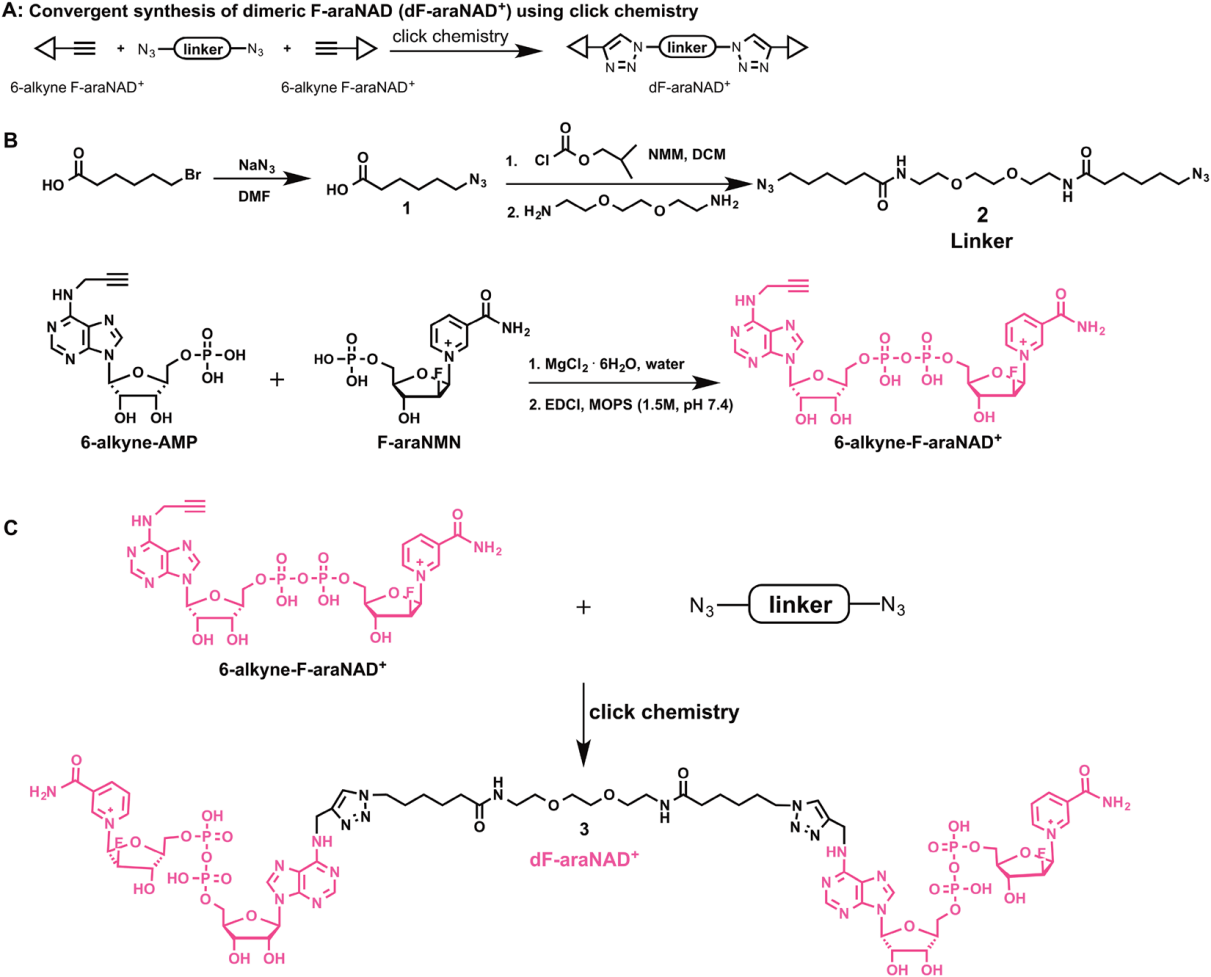
Synthesis of dimeric F-araNAD^+^ (dF-araNAD^+^). (**A**) The strategy used for convergent synthesis of dF-araNAD^+^ using click chemistry. (**B**) Synthesis of the linker and 6-alkyne-F-araNAD^+^ coupling reaction. (**C**) Synthesis of dF-araNAD^+^ using click chemistry, showing molecular structure of the small molecule.

To induce CD38 homodimerization, our approach was to synthesize a dimer of the small molecule arabinosyl-2′-fluoro-2′-deoxy NAD^+^ (F-araNAD^+^), a suicide substrate of CD38^32,33^. The synthesis involved conjugating two 6-alkyne-F-araNAD^+^ molecules via a diazido linker using the copper-catalysed Huisgen 1,3-dipolar cycloaddition between the alkyne and the azide functional groups, commonly known as click chemistry (Fig. 1A)^34^. The synthesis of 6-alkyne-F-araNAD^+^ followed a previous report, with the exception of the phosphate coupling reaction between 6-alkyne-AMP and a fluorinated nicotinamide mononucleotide, arabinosyl-2′-fluoro-2′-deoxy nicotinamide mononucleotide (F-araNMN)^35^. The modified coupling involved preparing the magnesium salts of 6-alkyne-AMP and F-araNMN, followed by the coupling reaction using N-(3-Dimethylaminopropyl)-N′-ethylcarbodiimide in MOPS buffer (1.5 M, pH 7.4) to afford 6-alkyne-F-araNAD^+^ (Fig. 1B). The diazido linker **2** was made by coupling two molecules of 6-azidohexanoic acid and one molecule of the diamino compound, 1,2-Bis(2-aminoethoxy)ethane (Fig. 1B). Finally, the diazido linker **2** was conjugated to 6-alkyne-F-araNAD^+^ via click chemistry to obtain the desired dimeric F-araNAD^+^ (dF-araNAD^+^) (Fig. 1C).

### dF-araNAD^+^ is able to induce covalent dimerization of CD38

**Figure 2:**
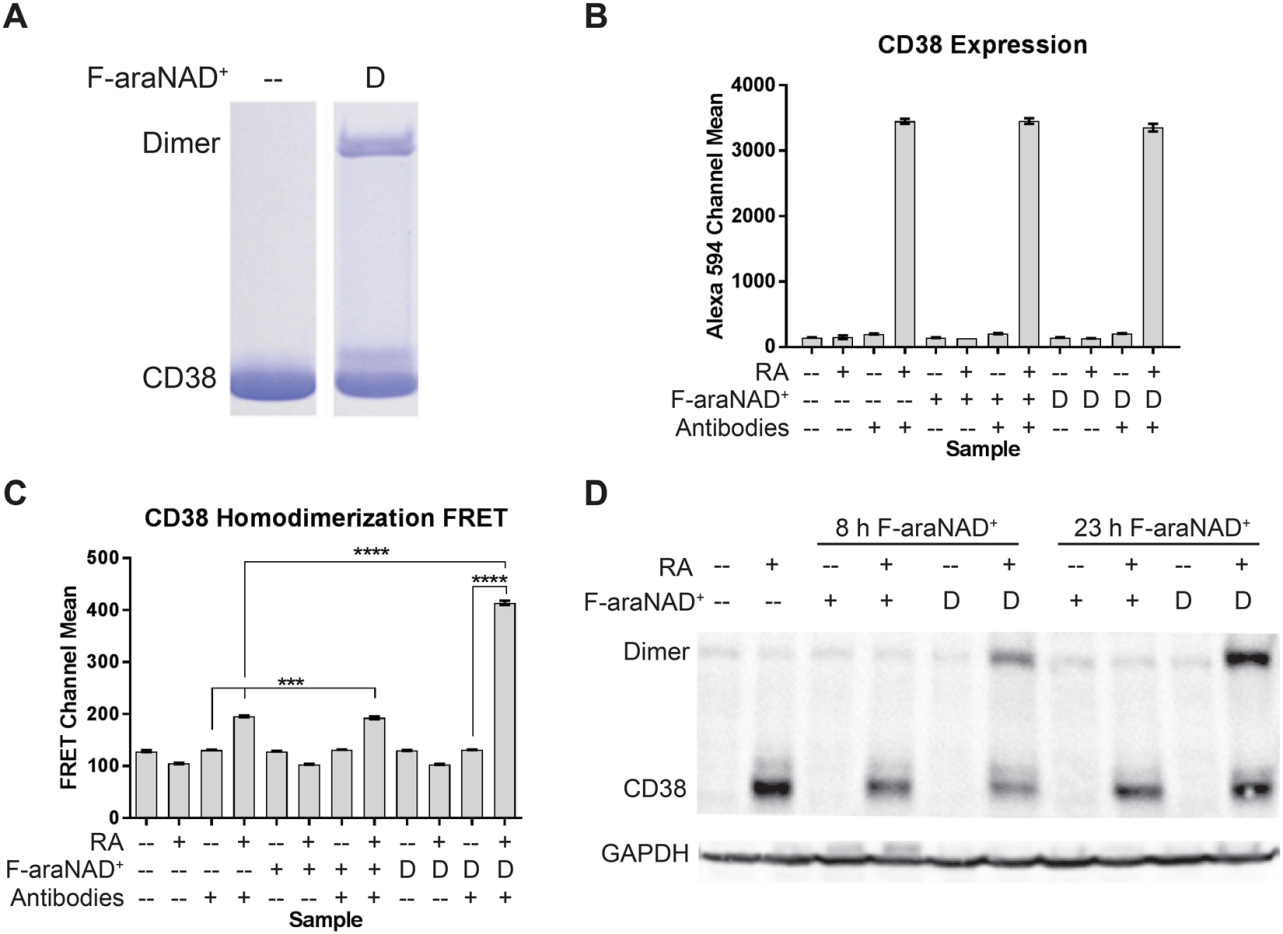
dF-araNAD^+^ induces CD38 homodimerization. (**A**) *In vitro* labelling of purified CD38. 5 μM purified CD38 was treated with dF-araNAD^+^ for 30 min as indicated. Reaction mixtures were resolved by SDS-PAGE and stained with Coomassie blue. (**B**) Mean fluorescence of HL-60 cells stained with Alexa Fluor 594-conjugated CD38 antibody (n = 3). HL-60 cells were cultured for 24 h with 1 μM RA as indicated. At 23 h, cells were treated with 1 μM F-araNAD^+^ (+) or dF-araNAD^+^ (D) as indicated. 1 × 10^6^ cells were put in PBS with Alexa Fluor 594-conjugated CD38 antibody as indicated and analysed by flow cytometry. Error bars indicate standard error of the mean (SEM). (**C**) FRET means (n = 3). HL-60 cells were cultured for 24 h with 1 μM RA as indicated. At 23 h, cells were treated with 1 μM F-araNAD^+^ (+) or dF-araNAD^+^ (D) as indicated. 1 × 10^6^ cells were put in PBS with Alexa Fluor 488- and 594-conjugated CD38 antibodies as indicated and analysed by flow cytometry. Error bars indicate SEM. ***p < 0.001; ****p < 0.0001. Two-way analysis of variance (ANOVA) using Tukey’s multiple comparisons test was used to determine significance. (**D**) Western blot showing covalent dimerization of CD38 when treated with dF-araNAD^+^. HL-60 cells were cultured with RA and treated with F-araNAD^+^ or dF-araNAD^+^ at 8 or 23 h as indicated and lysate was collected at 24 h. 25 μg of lysate per lane was run. Membrane images for each protein are cropped to show only the bands of interest.

We next examined whether dF-araNAD^+^ was able to induce dimerization of CD38. We first tested whether dF-araNAD^+^ could dimerize purified CD38 protein *in vitro*. As shown in Fig. 2A, following a 30 min incubation period, dF-araNAD^+^ was able to induce a degree of dimerization of purified CD38.

We then used FRET^36^ to detect CD38 dimerization in HL-60 cells. We directly conjugated monoclonal antibodies against CD38 with either Alexa Fluor 488 or 594 and, after determining that treatment with either F-araNAD^+^ or dF-araNAD^+^ did not alter CD38 expression (Fig. 2B), we performed the FRET measurement via flow cytometry (Fig. 2C). Untreated samples displayed background levels of fluorescence in the FRET channel. Samples with no CD38 antibodies are included to show that F-araNAD^+^ and dF-araNAD^+^ do not have background fluorescence. RA treatment induced CD38 expression and the acceptor fluorescence mean increased. When F-araNAD^+^ was added, there was no effect on the acceptor fluorescence. When dF-araNAD^+^ was added, however, the acceptor fluorescence mean increased. Therefore, in the presence of dF-araNAD^+^, the donor and acceptor fluors were closer, supporting that dF-araNAD^+^ increased the dimerization of CD38. Thus, while some CD38 homodimerization may be present after RA treatment alone, treatment with dF-araNAD^+^ increases the degree of CD38 homodimerization. This indicates that not all available CD38 was homodimerized before addition of dF-araNAD^+^.

Since dF-araNAD^+^ functions via covalent interactions, we also assessed and confirmed CD38 dimerization in HL-60 cells via western blot^32,33,35^. HL-60 cells that had been treated with RA to induce CD38 expression were treated with dF-araNAD^+^ at 8 h or 23 h and harvested at 24 h. In both cases, a band corresponding to twice the molecular weight of CD38 was present in the samples treated with RA and dF-araNAD^+^ (Fig. 2A and D). These results were consistent with those of the purified CD38 and FRET experiments, indicating that dF-araNAD^+^ induced dimerization of CD38.

### dF-araNAD^+^ does not affect RA-induced differentiation

In order to assess the effects of CD38 dimerization on RA-induced differentiation, we analysed several myeloid lineage differentiation markers over the course of a 72 h treatment. Since a previous report demonstrated that CD38 catalytic activity has no influence on RA-induced differentiation, we were not concerned about blocking the active site^30^. We measured CD38 surface expression at 24 h and cell density (i.e., population growth), CD11b surface expression, and G_1_/G_0_ cell cycle arrest every 24 h. HL-60 cell cultures were initiated with or without RA at 0 h, and F-araNAD^+^ or dF-araNAD^+^ was added to flasks as indicated at 8 h or 23 h when CD38 expression was significantly induced. This represented testing for both early and late potential dimerization effects.

**Figure 3:**
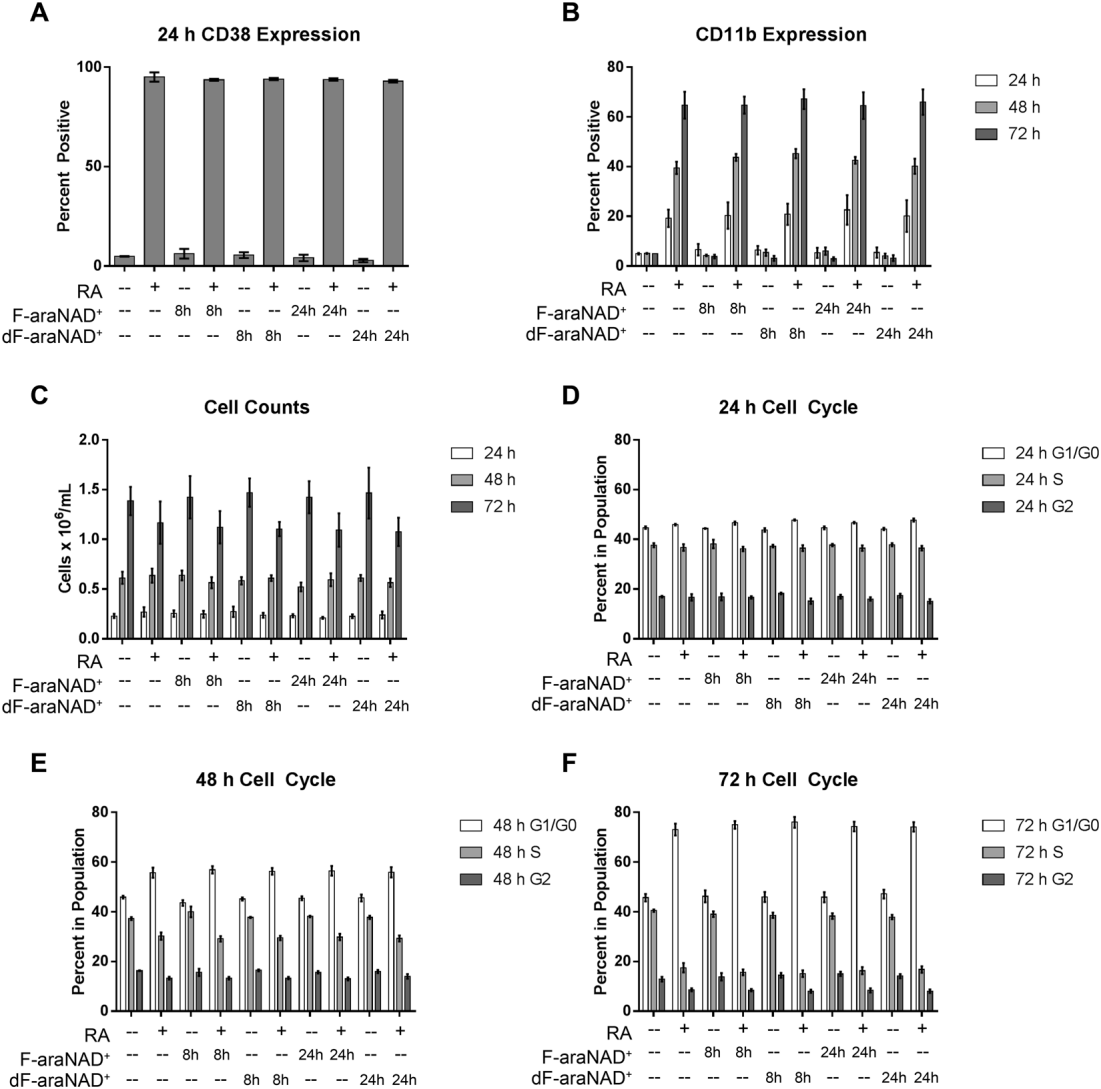
Induction of CD38 homodimerization does not phenotypically affect RA-induced differentiation. (**A**) HL-60 cells were cultured for 24 h with 1 μM RA as indicated and membrane CD38 was analysed using flow cytometry. Gating to discriminate positive cells was set to exclude 95% of untreated controls. 1 μM F-araNAD^+^ or dF-araNAD^+^ was added at 8 or 24 h as indicated. (**B**) Membrane CD11b was analysed using flow cytometry at 24, 48, and 72 h. (**C**) Cell density was measured at 24, 48, and 72 h using a haemocytometer and 0.2% Trypan Blue exclusion staining. (**D**–**F**) Cell cycle distribution, showing the percentage of cells in G_1_/G_0_, S, G_2_/M, was analysed using flow cytometry with propidium iodide staining at 24, 48, and 72 h. Error bars indicate SEM.

Expression levels of the differentiation markers varied as a function of RA, but not F-araNAD^+^ or dF-araNAD^+^, treatment (Fig. 3). As expected, with RA treatment, CD11b and CD38 expression increased, the proportion of cells in G_1_/G_0_ increased, and cell population growth was retarded compared to control cells not treated with RA. Addition of F-araNAD^+^ or dF-araNAD^+^ at either time point, however, had no effect on the induced expression of any of these markers.

### Characterization of CRISPR/Cas9-mediated CD38 disruption

Using the CRISPR/Cas9 system, we generated three CD38-targeted cell lines, CRISPR 1, 2, and 3, to test whether CD38 is required for RA-induced differentiation. Using the E-CRISP design tools, we targeted sites as early as possible along the gene to perform knockouts^37^.

**Figure 4:**
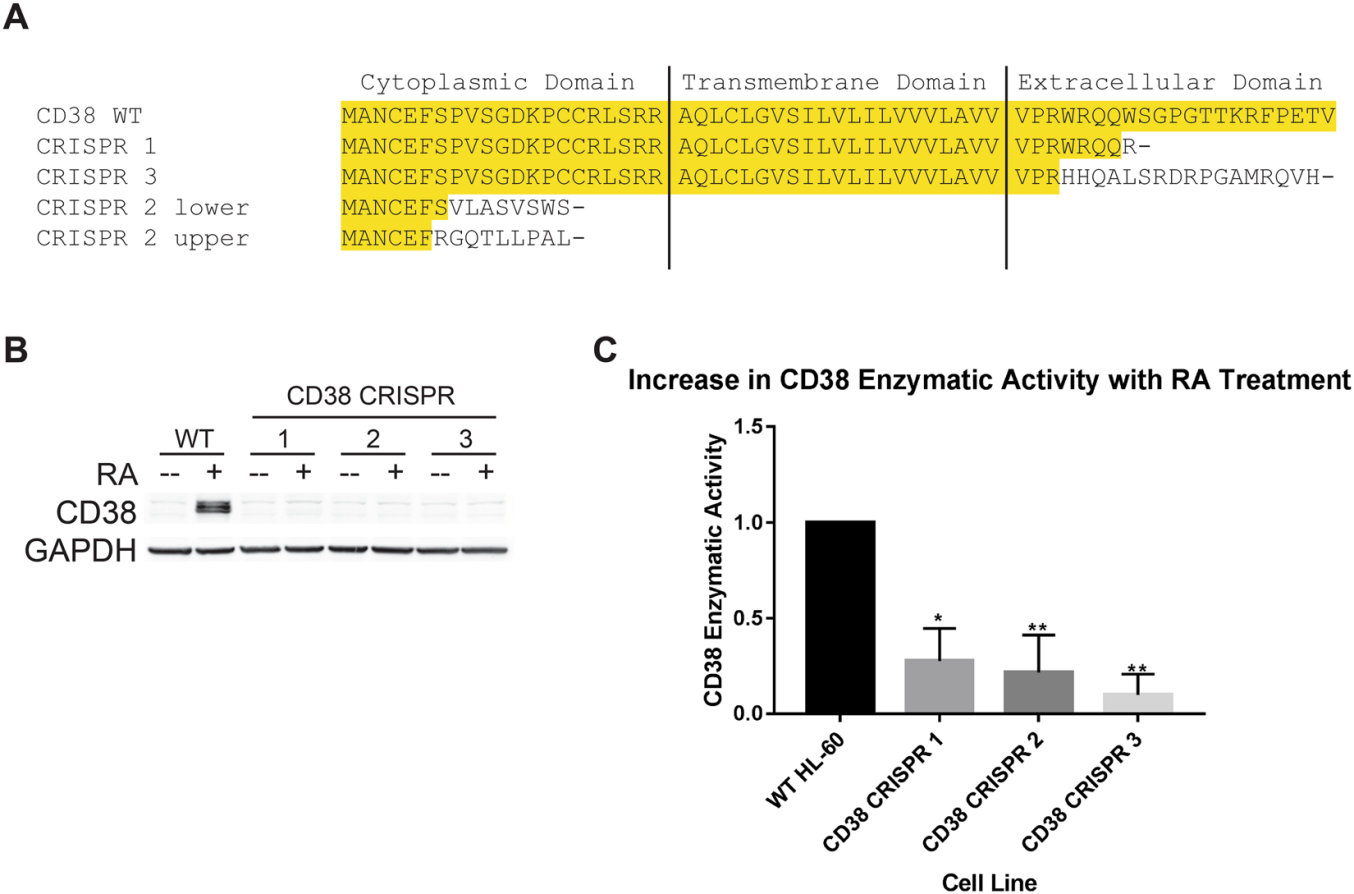
Disruption of CD38 using the CRISPR/Cas9 system. (**A**) Sequence alignment comparing the CD38 amino acid sequence of each CRISPR-derived cell line, up to the first stop codon, to wild-type CD38. Highlight denotes sequence matching wild-type CD38. The CRISPR 2 line is a mixed population expressing two different CD38 truncations. (**B**) Western blot of CD38. Wild-type and CRISPR-derived cell lines were treated with 1 μM RA as indicated for 48 h and whole cell lysate was collected. 25 μg of lysate per lane was run. Membrane images for each protein are cropped to show only the band of interest. (**C**) CD38 enzymatic activity assay. Wild-type and CRISPR cell lines were cultured for 48 h with 1 μM RA as indicated to stimulate CD38 expression and evaluated for cGDPR production via an NGD^+^ assay (n = 4). The difference in fold increase between RA treated and untreated samples was taken for each cell line and normalized to a control value of 1 in wild-type cells. Error bars indicate SEM. *p < 0.05; **p < 0.01. One-way ANOVA using Tukey’s multiple comparisons test was used to determine significance. The raw data NGD^+^ catabolism curves are shown in Supplementary Information.

**Figure 5:**
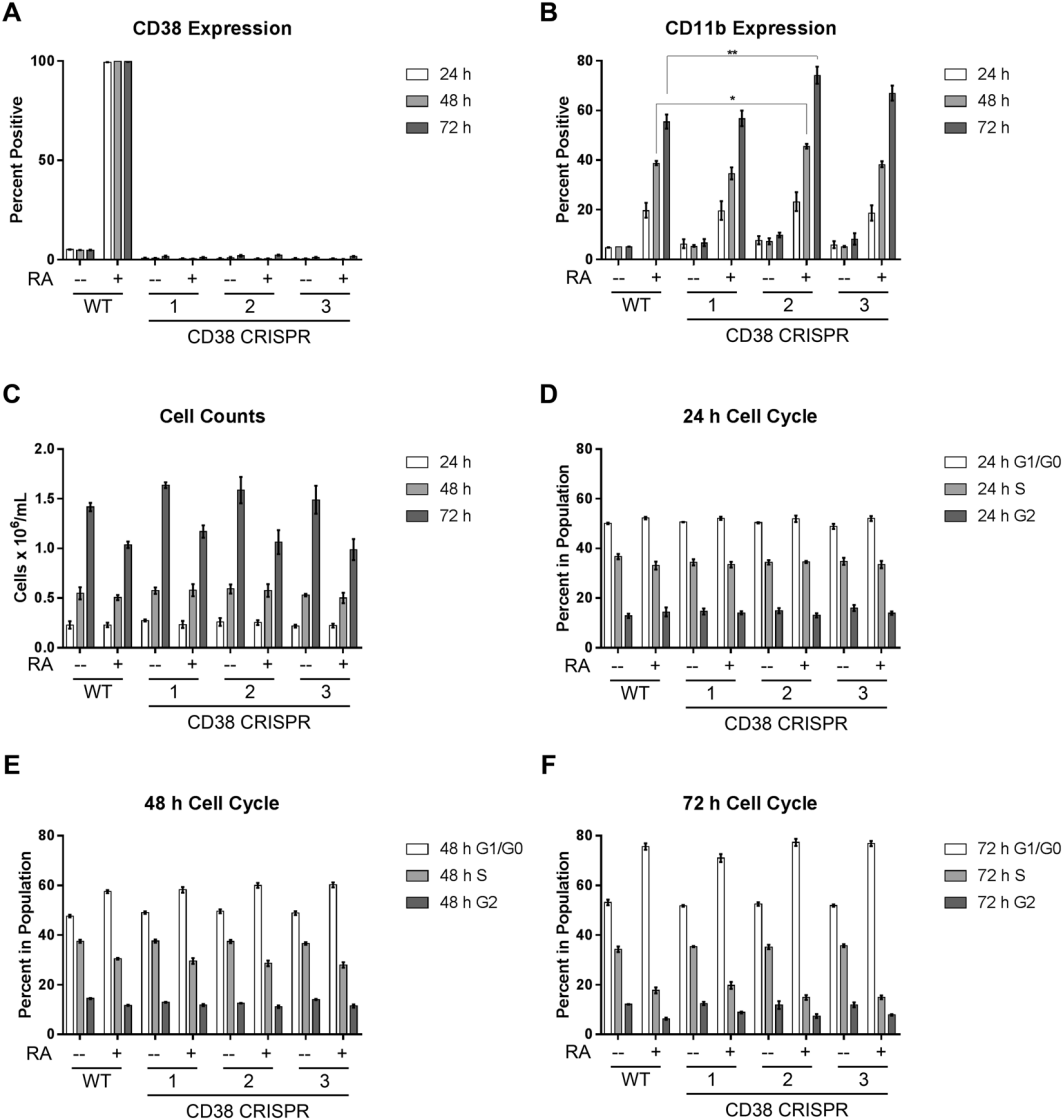
CRISPR/Cas9-mediated disruption of CD38 does not phenotypically affect RA-induced differentiation. (**A**) Wild-type and CRISPR-derived cell lines were cultured for 72 h with 1 μM RA as indicated and membrane CD38 expression was evaluated by flow cytometry at 24, 48, and 72 h. Gating to discriminate positive cells was set to exclude 95% of untreated wild-type cells. (**B**) Membrane CD11b expression was analysed at 24, 48, and 72 h using flow cytometry. *p < 0.05; **p < 0.01. Two-way ANOVA using Tukey’s multiple comparisons test was used to determine significance. (**C**) Cell density was measured at 24, 48, and 72 h using a haemocytometer and 0.2% Trypan Blue exclusion staining. (**D**–**F**): Cell cycle distribution was analysed using flow cytometry with propidium iodide staining at 24, 48, and 72 h. Error bars indicate SEM.

To ensure successful disruption, CD38 levels were measured by both flow cytometry and by western blot following 48 h treatment with RA. We detected no CD38 expression in any of the pooled cell lines (Figs 4B and 5A). We further characterized CD38 expression in the CRISPR-derived cell lines using RT-PCR (Fig. 4A). Comparing wild-type CD38 with the truncated forms, CRISPR 1 and 3 both lack nearly the entire extracellular domain, but retain the cytoplasmic and transmembrane domains (Fig. 4A). CRISPR 1 retains the N-terminal 49 amino acids before a substitution at position 50 and a premature stop codon. CRISPR 3 has a CD38 product of 62 amino acids before a premature stop codon, with the first 45 amino acids of wild-type CD38 conserved (Fig. 4A.). CRISPR 2 is a mixed population, as we saw two distinct bands after performing RT-PCR. Sequencing both bands, however, revealed that the CD38 truncations were similar – both lacked the vast majority of the protein, with only the first six or seven amino acids of CD38 left (Fig. 4A.). Both had premature stop codons after 15 amino acids (Fig. 4A).

The extracellular domain of CD38 contains its enzymatically active domain, and since we largely deleted this, we anticipated a loss of RA-induced enzymatic activity with the CRISPR mutants. We confirmed this using the nicotinamide guanine dinucleotide (NGD^+^) assay (Fig. 4C), which detects ADP-ribosyl cyclase activity. While CD38 is a major source of this activity, CD157 also possesses this catalytic activity^38^. HL-60 cells normally express both CD38 and CD157 at very low levels, but they are upregulated following RA treatment. We calculated the fold increase in cyclic GDP ribose (cGDPR) production from RA-treated to untreated cells for each mutant line and normalized to wild-type HL-60 levels. In each of the CRISPR-derived lines, RA-induced production of cGDPR was significantly reduced, further confirming the disruption of CD38.

### CD38 disruption does not affect RA-induced differentiation

We evaluated the effects of CD38 disruption on RA-induced differentiation as we did with induced CD38 dimerization. We measured cell density, CD11b, CD38, and G_1_/G_0_ cell cycle arrest over the course of a 72 h RA treatment. For both wild-type and CRISPR-derived cell lines, RA treatment induced growth retardation, upregulation of CD38 and CD11b expression, and G_1_/G_0_ accumulation (Fig. 5). The responses of the CRISPR-derived knockouts were not significantly different from wild-type cells except for the CRISPR 2 cell line, which displayed increased levels of CD11b at 48 and 72 h after RA treatment (Fig. 5B).

### CD38 disruption does not affect prominent signalling proteins associated with RA-induced differentiation

**Figure 6:**
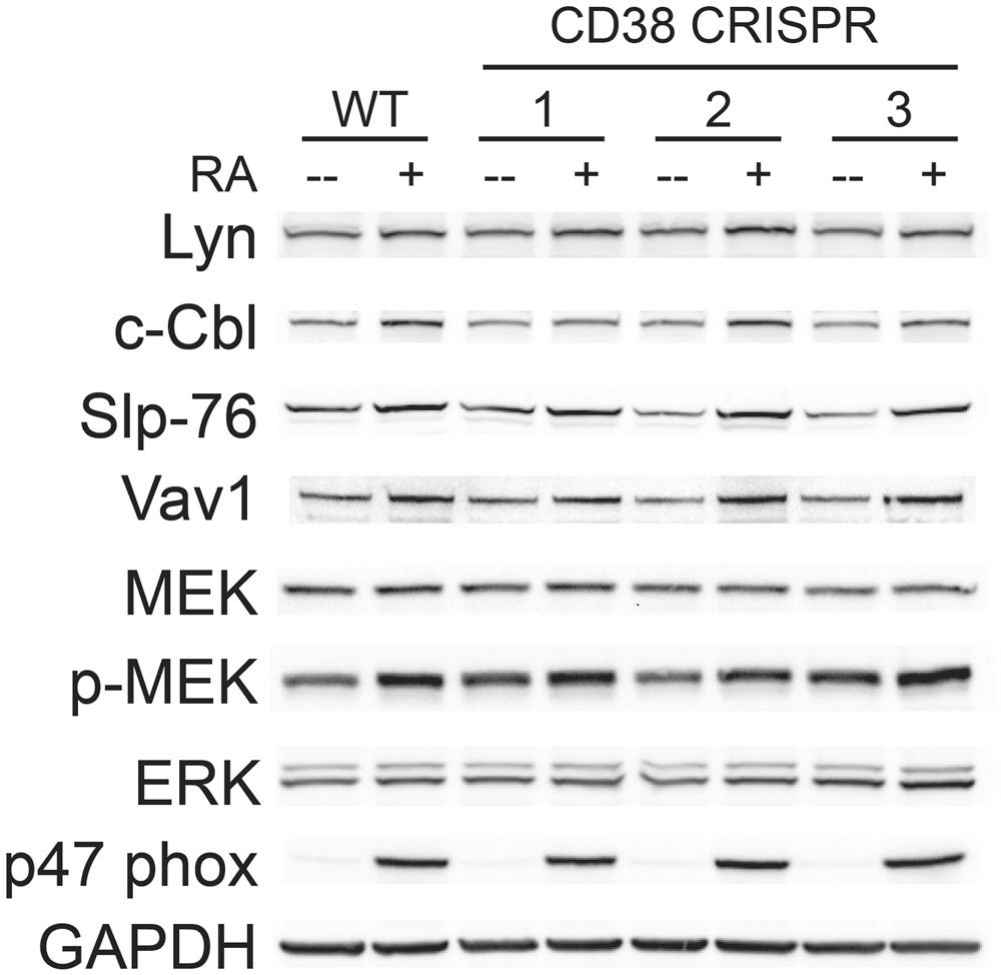
CRISPR/Cas9-mediated disruption of CD38 does not affect signalling proteins known to be involved in RA-induced differentiation. Wild-type and CD38 CRISPR cell lines were cultured for 48 h with 1 μM RA as indicated and whole cell lysate was collected. 25 μg of lysate per lane was run. Western blots of PAGE-resolved lysates from wild-type cells and CRISPR 1, 2, and 3 cell lines were probed for the indicated proteins, where GAPDH is a loading control. Membrane images for each protein are cropped to show only the band of interest.

Since an ensemble of signalling molecules propels differentiation, we determined whether any RA-response associated signalling proteins were affected by loss of the majority of CD38. c-Cbl, Lyn, Slp-76, and Vav1, which have been shown to interact with CD38, were of particular interest^19–21^. We treated wild-type HL-60s, as well as the CRISPR-derived cell lines, with or without RA for 48 h, collected lysates, and analysed them by western blot (Fig. 6). For all the proteins we analysed, we observed that the RA-induced effects were similar for wild-type and CRISPR-derived cells (Fig. 6). This corroborates the previously described results for phenotypic shift of the cells. Levels of the signalling proteins Lyn, c-Cbl, Slp-76, Vav1, and phospho-MEK increased similarly with RA treatment for wild-type and CRISPR-derived lines (Fig. 6). RA treatment induced expression of a component of the NAD^+^ phosphatase oxidase complex, p47 phox, in all cell lines as well (Fig. 6). MEK and ERK levels remained stable (Fig. 6).

## Discussion

While a previous report indicated that CD38 is found as homodimers on the cell surface of murine B lymphocytes, our results suggest that dF-araNAD^+^ is able to induce an additional degree of CD38 homodimerization in HL-60 cells^31^. This increased CD38 homodimerization did not affect RA-induced differentiation. We must note, however, that there are several alternative explanations for why dF-araNAD^+^ did not affect RA-induced differentiation. It is possible that the induced CD38 dimer is not in the correct orientation to initiate downstream signalling or that high-order oligomerization other than dimerization is needed.

Additionally, we do not know whether a particular degree of dimerization is required for signalling. One of the best studied transmembrane receptors, epidermal growth factor receptor (EGFR), is typically found in a 3:7 ratio of dimer to monomer under resting conditions and a 1:1 ratio when its cognate ligand, EGF, is bound^39^. The western blot of relative abundance of CD38 monomer and dimers, although not quantitative, appears consistent with this range of receptor dimerization. *In vitro* dimerization of EGFR with EGF and activation of EGFR tyrosine kinase activity are coincident events, but transmembrane receptor dimer formation and downstream signalling are not always coupled^40–42^. It is experimentally intractable to test every possibility such as the dimer to monomer ratios, relation between dimer formation and downstream signalling, required orientation, and potential high-order oligomerization for CD38. Thus, we took a simpler alternative approach to study the role of CD38 in RA-induced differentiation: We genetically disrupted endogenous CD38 using the CRISPR/Cas9 system to probe for effects of losing the protein.

Results from the CRISPR/Cas9-mediated CD38 truncations indicate that the extracellular domain of CD38 is not necessary for RA-induced differentiation. CRISPR 1 and CRISPR 3 possess only seven and three amino acids, respectively, of the extracellular domain of CD38, while CRISPR 2 has none, yet the phenotypic response to RA treatment is largely unaltered. Because the enzymatic activity of CD38 is dependent on the extracellular domain, specifically E226, we also conclude that this enzymatic activity is not necessary for RA-induced differentiation, consistent with previously reported results^30^.

CRISPR 2 is a mixed population expressing two different short peptides of CD38, both of which are 15 amino acids long, with the first six or seven amino acids conserved. Phenotypic markers for RA-induced differentiation in the CRISPR 2 line were equal to or, in the case of CD11b, better than wild-type HL-60 cells. The transmembrane portion and much of the cytoplasmic domain are thus also not required for RA-induced differentiation. We cannot rule out the importance of the remaining short peptide; however, the weight of the evidence suggests that CD38 is not necessary for RA-induced myeloid differentiation. This conclusion can be integrated with that of an earlier report showing that siRNA- and morpholino-mediated knockdown of CD38 compromised RA-induced differentiation of HL-60 cells^18^. That report tested differentiation using only one assay, nitroblue tetrazolium reduction, a marker for inducible reactive oxidative metabolism. Occurrence of mature myeloid cells is commonly measured by either the CD11b cell surface marker or by functional differentiation evidenced by 12-*O*-tetradecanoylphorbol-13-acetate-inducible superoxide production. It is also betrayed by occurrence of G_1_/G_0_ arrest. While Munshi *et al*. measured superoxide production, which is a functional marker for mature cells, we measured CD11b, which is a widely accepted surface marker for mature cells, as well as G_0_ arrest and expression of p47 phox, a signature component of the superoxide production machinery. Evidenced by the cell surface and cell cycle indicators, there was cell maturation and cell cycle arrest following treatment of the CRISPR-derived cell lines with RA. Induced expression of p47 phox was also not compromised. Synthesizing our data and theirs, it is possible that while some aspect of the NADPH machinery generating superoxide is CD38 dependent, the expression of the mature cell surface marker is not, nor is cell cycle arrest and nor is the induced expression of p47 phox. It is potentially also noteworthy that crippling CD38 may affect NAD^+^ metabolism so that it adversely affects the function of NADPH machine and ergo superoxide production. Without being able to rule this out, superoxide production may not be a reliable marker of differentiation when NAD^+^ metabolism is subject to manipulation. Hence, unless a 6-amino acid fragment of the cytoplasmic tail can account for any differentiation-driving action of the entire CD38 receptor, it appears that CD38 is not necessary for the signalling or differentiation attributed to RA.

In summary, our data show that despite the evidence suggesting that CD38 is a driver of RA-induced differentiation, neither induction of dimerization nor deletion of the vast majority of the protein affected the differentiation process. It appears that CD38 is ergo not necessary for RA to induce differentiation, even though enhancing its expression can enhance signalling and differentiation. A full understanding of its function remains to be determined. Given that CD38 is upregulated so prominently and so early in response to RA treatment, it is possible that it may play an important role in the function of differentiated cells. HL-60 cells differentiate into neutrophil-like cells after RA treatment. Consistent with this, CD38 knockout mice are known to have immune defects^43^. However, exactly how CD38 affects the function of immune cells remains to be elucidated.

## Methods

### Cell culture

Reagents, unless specified otherwise, were purchased from commercial suppliers in the highest purity available and used as supplied. HL-60 human myeloblastic leukaemia cells and stable transfectant cell lines (CRISPR 1, CRISPR 2, CRISPR 3) were cultured in RPMI 1640 supplemented with 5% heat-inactivated foetal bovine serum (GE Healthcare, Chicago, IL) and 1% antibiotic/antimycotic (Thermo Fisher Scientific, Waltham, MA) in a 5% CO_2_ humidified atmosphere at 37 °C. RA (Sigma, St. Louis, MO) was solubilized in absolute ethanol. A final concentration of 1 µM was used. Arabinosyl-2′-fluoro-2′-deoxy NAD^+^ (F-araNAD^+^) and dimeric F-araNAD^+^ (dF-araNAD^+^) were used at final concentrations of 1 µM.

### Antibodies and reagents

PE-conjugated CD38 (clone HIT2, catalogue number 555460, lot 5027616), APC-conjugated CD11b (clone ICRF44, catalogue number 550019, lot 5051946), CD38 for western blotting (clone 22/CD38, catalogue number 611114, lot 3295551), and CD38 antibodies for direct conjugation to Alexa Fluors (clone HIT2, catalogue number 555458, lot 4080720) were purchased from Becton Dickinson (Franklin Lakes, NJ). GAPDH (clone D16H11, catalogue number 5174 S, lot 6), Lyn (catalogue number 2732 S, lot 4), Slp76 (catalogue number 4958 S, lot 2), Vav1 (catalogue number 2502 S, lot 2), total MEK 1/2 (catalogue number 9122 L, lot 14), phospho-MEK 1/2 (clone 41G9, catalogue number 9154 S, lot 14), total ERK 1/2 (clone 137F5, catalogue number 4695 S, lot 14), p47 phox (clone D21F6, catalogue number 4301 S, lot 1), HRP-linked anti-mouse (catalogue number 7076 S, lot 32), and HRP-linked anti-rabbit (catalogue number 7074 S, lot 26) antibodies were purchased from Cell Signaling Technologies (Danvers, MA). Anti-c-Cbl (clone C-15, catalogue number sc-170, lot H0414) was purchased from Santa Cruz Biotechnology (Santa Cruz, CA). Alexa Fluor 488 and 594 succinimidyl esters, Stbl3 competent cells, and slide-a-lyzer MINI 20 kDa molecular weight cutoff (MWCO) dialysis units were purchased from Thermo Scientific (Waltham, MA). The eCRISP small guide RNA (sgRNA) tool was used to select sgRNA specific to CD38 and oligos were purchased from IDT (Coralville, IA)^41^.

### Dimerization of purified CD38

CD38 was expressed and purified as previously described^44,45^. 5 µM purified CD38 was incubated for 30 min at 37 °C with or without 20 µM dF-araNAD^+^ in 10 µL reaction buffer (25 mM HEPES, 50 mM NaCl, pH 7.4) and then mixed with 2 µL 6x protein loading buffer. Samples were heated at 100 °C for 7 min, resolved by SDS-PAGE, and stained with Coomassie blue.

### Direct conjugation of Alexa Fluors to primary antibodies

Conjugation of Alexa Fluor succinimidyl esters to CD38 primary antibodies was performed using the manufacturer’s protocol. Briefly, 30 µL 0.75 M sodium bicarbonate, pH 8.3, was added to 200 µL CD38 antibody. 1 mg of Alexa Fluor was dissolved in 100 µL DMSO, and 20 µL Alexa Fluor 488 or 594 was added to one vial of primary antibody while vortexing. The tubes were then shaken at room temperature for 1 h at 200 RPM. 20 µL 1.5 M hydroxylamine, pH 8.5, was then added to each tube and they were shaken for an additional 1 h. Finally, the conjugated antibodies were dialyzed in 2 L of PBS in the dark at room temperature for 2 h.

### Fluorescence resonance energy transfer

HL-60 cells were cultured for 24 h with 1 µM RA as indicated. At 23 h, F-araNAD^+^ or dF-araNAD^+^ was added to appropriate flasks. At 24 h, 1 × 10^6^ cells from each sample were centrifuged at 700 RPM for 5 min. Samples were washed with PBS twice before being resuspended in 200 µL PBS containing 5 µL of a 1:1 mixture of Alexa Fluor 488- and 594-conjugated CD38 antibodies. Samples were incubated for 1 h at 37 °C in the dark. Samples were analysed using a Becton Dickinson FACS Aria III SORP (San Jose, CA). To measure the FRET signal, a 488 nm laser line was used to excite Alexa Fluor 488, which in turn excited Alexa Fluor 594; emission from Alexa Fluor 594 was collected through a 600 nm longpass dichroic mirror and a 610/20 nm bandpass filter. Unstained controls and controls stained with Alexa Fluor 488-conjugated CD38 or Alexa Fluor 594-conjugated CD38 alone were used to set compensation values.

### Western blot analysis

Cells were pelleted, washed twice with PBS, and lysed with ice cold M-PER Mammalian Protein Extraction Reagent (Pierce, Rockford, IL) with protease and phosphatase inhibitor cocktails (Sigma, St. Louis, MO). Samples were incubated overnight at −80 °C and debris was pelleted. Protein concentration was determined using the Pierce BCA Protein Assay according to the manufacturer’s protocol. Lysate was subjected to standard SDS-PAGE, using 25 µg of lysate per lane under denaturing conditions. Membranes were blocked with 5% dry nonfat milk in PBS and probed with antibodies described above. Images were captured on a Bio-Rad VersaDoc MP 5000 and analysed with Quantity One software (Hercules, CA).

### Flow cytometric phenotypic analysis

Immunostaining for CD11b and CD38 was performed as previously described and analysed using a Becton Dickinson LSR II flow cytometer (San Jose, CA)^46^. Gating was set to exclude 95% of the untreated wild-type HL-60 samples. Propidium iodide (PI) cell cycle analysis was performed as previously described^46,47^.

### Generation of stable transfectants

CD38 was targeted for CRISPR/Cas9-mediated disruption using sgRNA sequences generated by the E-CRISP tool^41^. Sequences were designed for use in the pLentiCRISPR v2 plasmid (Addgene #52961) (Addgene, Cambridge, MA) and obtained from IDT (Coralville, IA). Three sgRNA sequences were used (CRISPR 1, CRISPR 2, and CRISPR 3) to minimize off-target effects. The sequences are: CRISPR 1 (F: 5′-CACCGCGCCAGCAGTGGAGCGGTCC-3′; R: 5′-AAACGGACCGCTCCACTGCTGGCGC-3′), CRISPR 2 (F: 5′-CACCGCAGGGTTTGTCCCCGGACAC-3′; R: 5′-AAACGTGTCCGGGGACAAACCCTGC-3′), and CRISPR 3 (F: 5′-CACCGCTCCACTGCTGGCGCCACCT-3′; R: 5′-AAACAGGTGGCGCCAGCAGTGGAGC-3′). These sequences were each cloned into pLentiCRISPR v2 following the depositor’s protocol.

Lentiviral particles were produced using 2.5 µg pMD2.g (Addgene #12259), 7.5 µg psPAX2 (Addgene #12260), and 10 µg pLentiCRISPR v2 with CRISPR 1, 2, or 3 inserted. These were co-transfected into HEK 293 T cells at roughly 50% confluence in 10 cm cell culture dishes with DMEM and 10% FBS using TransIT-LT1 transfection reagent (Mirus, Madison, WI) according to the manufacturer’s protocol in a 3:2 ratio of reagent (µL) to plasmid (µg). After 48 h, media containing viral particles was collected and 5 mL of additional media was added to the dishes for 24 h until final collection. The 15 mL total viral media was concentrated using Amicon Ultra (Millipore, Billerica, MA) centrifugal filters with 30 kDa MWCO. Concentrated viral media was stored at −80 °C until use.

Transduction of HL-60 cells with the lentiviral particles was performed in 6-well plates. 100 µL concentrated viral particles was added to 5 × 10^4^ cells in 1 mL RPMI 1640 with 5% heat-inactivated FBS. After 72 h, transduced cells were put into 10 mL flasks and cultured in RPMI 1640 with 5% FBS and selected for 3 weeks in 400 ng/mL puromycin. The cell lines were evaluated for CD38 expression following 24 h treatment with 1 µM RA. In all cases, while the majority did not upregulate CD38 in response to RA treatment, there was a population of 15–30% that expressed CD38 levels comparable to wild-type HL-60 cells. Thus, a single cell sort into a 96-well plate was performed on each cell line using a Becton Dickinson FACS Aria III SORP. Upon confluence, each well was transferred to a 24-well plate and RA-induced CD38 expression was assessed as before. Several wells per cell line which did not express CD38 were pooled and designated CRISPR 1, CRISPR 2, and CRISPR 3 according to the sgRNA used.

### mRNA isolation and sequencing

Total RNA was extracted from CRISPR 1, CRISPR 2, and CRISPR 3 cell lines using a Qiagen RNeasy Plus Mini kit following the manufacturer’s protocol (Hilden, Germany). RT-PCR was performed using Invitrogen SuperScript III One Step RT-PCR with Platinum Taq (Invitrogen, Carlsbad, CA) on a PTC-100 thermocycler from MJ Research (Waltham, MA). Forward (5′-AGAAGGGGAGGTGCAGTTTC-3′) and reverse (5′-TGTTGCAAGGTACGGTCTGA-3′) primers for CD38 were purchased from IDT (Coralville, IA). Products were separated on a 1.5% acrylamide gel and appropriate bands were excised and purified with a Qiagen QIAquick Gel Extraction Kit according to the manufacturer’s protocol. Because the lines were established from several populations, there were two bands corresponding to slightly different CD38 mutants in the CRISPR 2 line, denoted upper and lower. DNA samples were sequenced at Cornell University’s Life Sciences Core Laboratory. DNA sequences were translated to amino acid sequences using ExPASY Translate and compared to the sequence of wild-type CD38.

### Detection of CD38 enzymatic activity

ADP-ribosyl cyclase activity was detected by fluorometric analysis of the NGD^+^ metabolic product cyclic GDP ribose (cGDPR) using a spectrofluorometer (Horiba Jobin Yvon FluoroMax3, NJ) as previously described^30^. Cells were cultured for 48 h treated with or without 1 µM RA to induce CD38 expression. 1 × 10^6^ cells were pelleted, washed twice with PBS, resuspended in 0.7 mL PBS, and treated with 100 µM NGD^+^ for 1 h with constant rotation at 37 °C. Cells were then pelleted and the supernatant was analysed for cGDPR. Readings at 300 nm excitation/410 nm emission were used to measure cGDPR content.

### Statistics

Statistics were analysed using GraphPad Prism version 7.02. One-way and two-way analysis of variance, as appropriate, with Tukey’s multiple comparisons test was used to determine significance. Error bars indicate mean ± standard error of the mean (SEM).

### Data availability

The datasets generated during and/or analysed during the current study are available from the corresponding author on reasonable request.

## Electronic supplementary material

Supplementary Information
