# Supplementary material for: Probing the requirement for CD38 in retinoic acid-induced HL-60 cell differentiation with a small molecule dimerizer and genetic knockout
Source: Sci Rep. 2017 Dec 12;7:17406. doi: 10.1038/s41598-017-17720-4 (PMC5727258; doi:10.1038/s41598-017-17720-4)
Supplement: Supplementary file 1 — Supplementary Information [file 41598_2017_17720_MOESM1_ESM.pdf]

Supplementary Information

Probing the requirement for CD38 in retinoic acid-induced HL-60 cell differentiation with a small molecule dimerizer and genetic knockout

Robert J. MacDonald[1], Jonathan H. Shrimp[2], Hong Jiang[2], Lu Zhang[2], Hening Lin[3], and Andrew Yen*[1]

[1]Department of Biomedical Sciences, Cornell University, Ithaca, NY 14853
[2]Department of Chemistry and Chemical Biology, Cornell University, Ithaca, NY 14853
[3]Howard Hughes Medical Institute, Department of Chemistry and Chemical Biology, Cornell University, Ithaca, NY 14853
*To whom correspondence should be addressed: Andrew Yen, 1-607-253-3354, ay13@cornell.edu

Table of Contents

General experimental methods--S2

Synthetic Scheme for dF-araNAD$^+$ ---S3

Synthesis of the linker ---S4

Synthesis of 6-alkyne-F-araNAD$^+$--S6

Synthesis of dF-araNAD$^+$---S7

Uncropped western blot images--S9

Raw NGD$^+$ catalysis curves---S11

General experimental methods. Reagents, unless specified otherwise, were purchased from commercial suppliers in the highest purity available and used as supplied. ^{1}H NMR was performed on INOVA 400/500/600 spectrometers, ^{13}C NMR was performed on INOVA 400 spectrometer, and 2D NMR was performed on INOVA 500/600 spectrometers. NMR data was analysed using MestReNova (version 8.1.1). ^{1}H NMR chemical shifts are reported in units of ppm relative to tetramethylsilane. ^{1}H NMR data are reported in the following manner: chemical shift (multiplicity, integration). LC-MS experiments were carried out on a Shimadzu HPLC LC20-AD and Thermo Scientific LCQ Fleet with a Sprite TARGA C18 column (40 × 2.1 mm, 5 μm, Higgins Analytical, Inc.) monitoring at 215 and 260 nm with positive or negative mode for mass detection. Solvents for LC-MS were water with 0.1% acetic acid (solvent A) and acetonitrile with 0.1% acetic acid (solvent B). Compounds were eluted at a flow rate of 0.3 mL/min with 0% solvent B for 2 min, followed by a linear gradient of 0% to 10% solvent B over 2 min, followed by a linear gradient of 10% to 100% solvent B over 5 min, and finally 100% solvent B for 1 min before equilibrating the column back to 0% solvent B over 1 min. Preparative HPLC experiments were done on Beckman Coulter System Gold 125p Solvent Module & 168 Detector with a TARGA C18 column (250 × 20 mm, 10 μm, Higgins Analytical, Inc.) monitoring at 215 and 260 nm. Solvents for prep HPLC were water with 0.1% trifluoroacetic acid (solvent A) and acetonitrile with 0.1% trifluoroacetic acid (solvent B). Compounds were eluted at a flow rate of 8.0 mL/min with solvent gradient described later for specific compounds.

Figure S1: Synthetic scheme for dF-araNAD$^+$

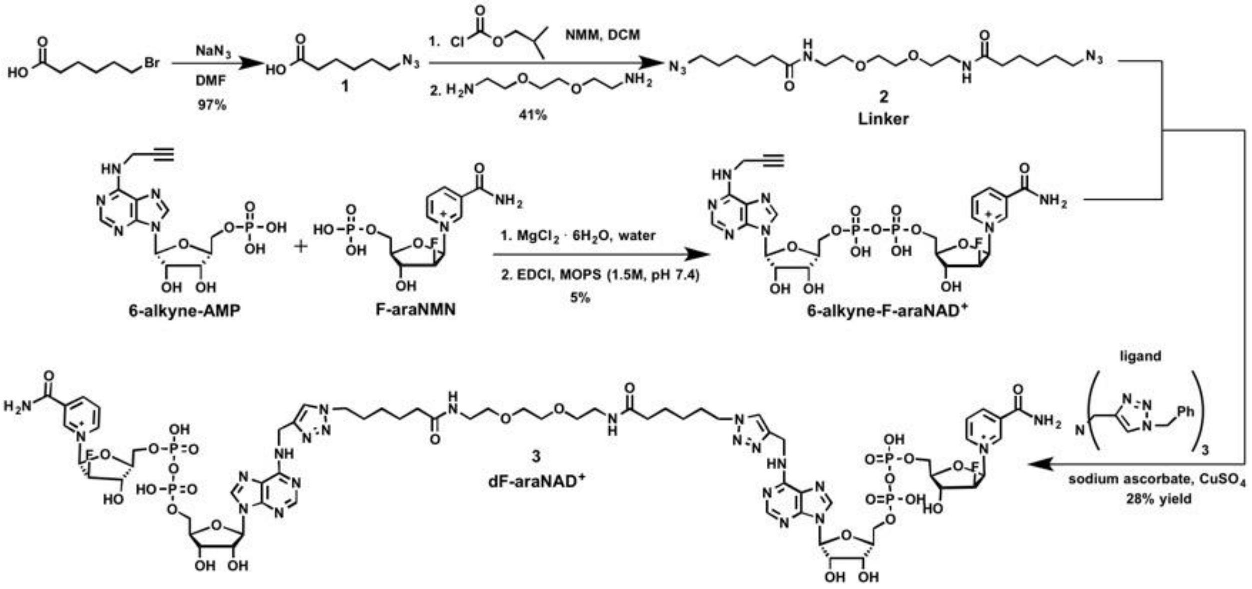

Synthesis of 6-azido hexanoic acid (1). To a solution of 6-bromohexanoic acid (1.48 g, 7.59 mmol, 1 eq) in 8.3 mL DMF in a round-bottom flask fitted with a stir bar was added sodium azide (1.0 g, 15.4 mmol, 2.0 eq) with stirring until all solid dissolved. Subsequently, the reaction was put into an 85°C oil bath and stirred. The reaction was monitored by LC-MS (260 nm) and by TLC. After 7.5 h, the reaction was diluted in DCM and washed using 0.1 M HCl (3 x 100 mL). The organic layers were dried over anhydrous Na_2SO_4, then concentrated on a rotary evaporator, and further dried under high vacuum. Compound **1** was obtained as a colourless liquid (1.167 g, 97% yield) and was used without further purification. R_f was 0.63 in 7:3 DCM:EtOAc with 0.5% acetic acid. LC-MS (ESI) calcd. for $C_6H_{10}N_3O_2^-$ [M^-] 156.08 and [$2(M^-)$] 312.16, obsd. 156.58 and 313.25.

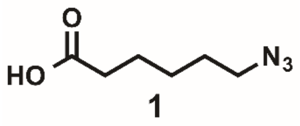

Figure S2: LC-MS of 6-azido hexanoic acid

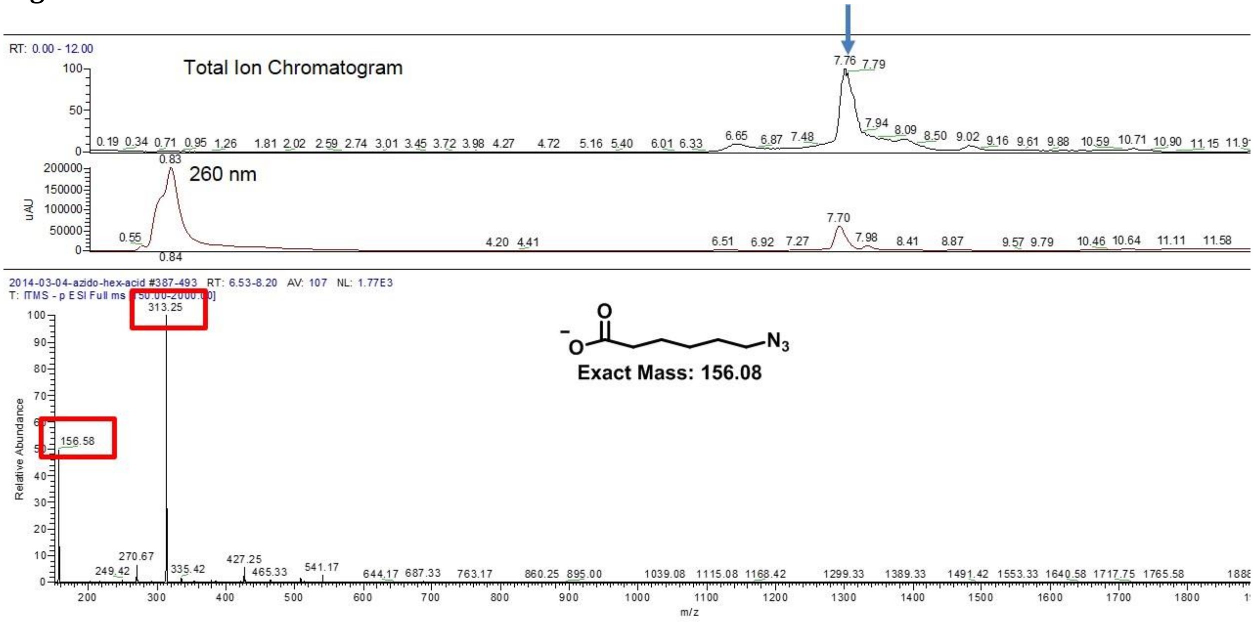

Synthesis of the linker (2). N-methylmorpholine (0.094 mL, 0.857 mmol, 2.7 eq) and isobutylchloroformate (0.112 mL, 1.15 mmol, 3.6 eq) were added to a solution of **1** (200 mg, 1.27 mmol, 4.0 eq) in 6.5 mL anhydrous DCM in a round-bottom flask fitted with a stir bar under nitrogen at 0°C. The homogenous solution was stirred for 30 min at 0°C. Subsequently, 1,2-Bis(2-aminoethoxy)ethane (47.1 mg, 0.318 mmol, 1.0 eq) was added. The homogenous solution was stirred for 45 min while under nitrogen at 0°C. The reaction was then warmed to rt and stirred overnight. Product **2** formation was confirmed by LC-MS. The reaction was diluted in DCM and washed with 0.1 M NaOH (2 x 50 mL) and water (1 x 50 mL) to remove excess compound **1**. The organic layer was dried over anhydrous Na_2SO_4, concentrated on a rotary evaporator, and further dried under high vacuum. Purification was done using silica gel flash chromatography (20:1 DCM:MeOH eluted the product). Compound **2** was obtained as a solid (55 mg, 41% yield). ^{1}H NMR (400 MHz, $CDCl_3$): δ 6.12 (s, 2H), 3.55 (s, 4H), 3.49 (t, J = 5.41 Hz, 4H), 3.38 (q, J = 5.21 Hz, 4H), 3.21 (t, J = 6.65 Hz, 4H), 2.14 (t, J = 7.19 Hz, 4H), 1.65 – 1.50 (m, 9H), 1.40 – 1.30 (m, 4H). LC-MS (ESI) calcd. for $C_{18}H_{34}N_8O_4$ $[(M+H)^+]$ 427.27, obsd. 427.25.

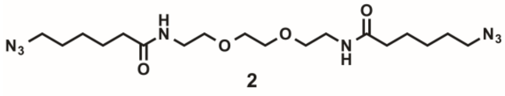

2
Linker

Figure S3: ^{1}H NMR of compound **2**

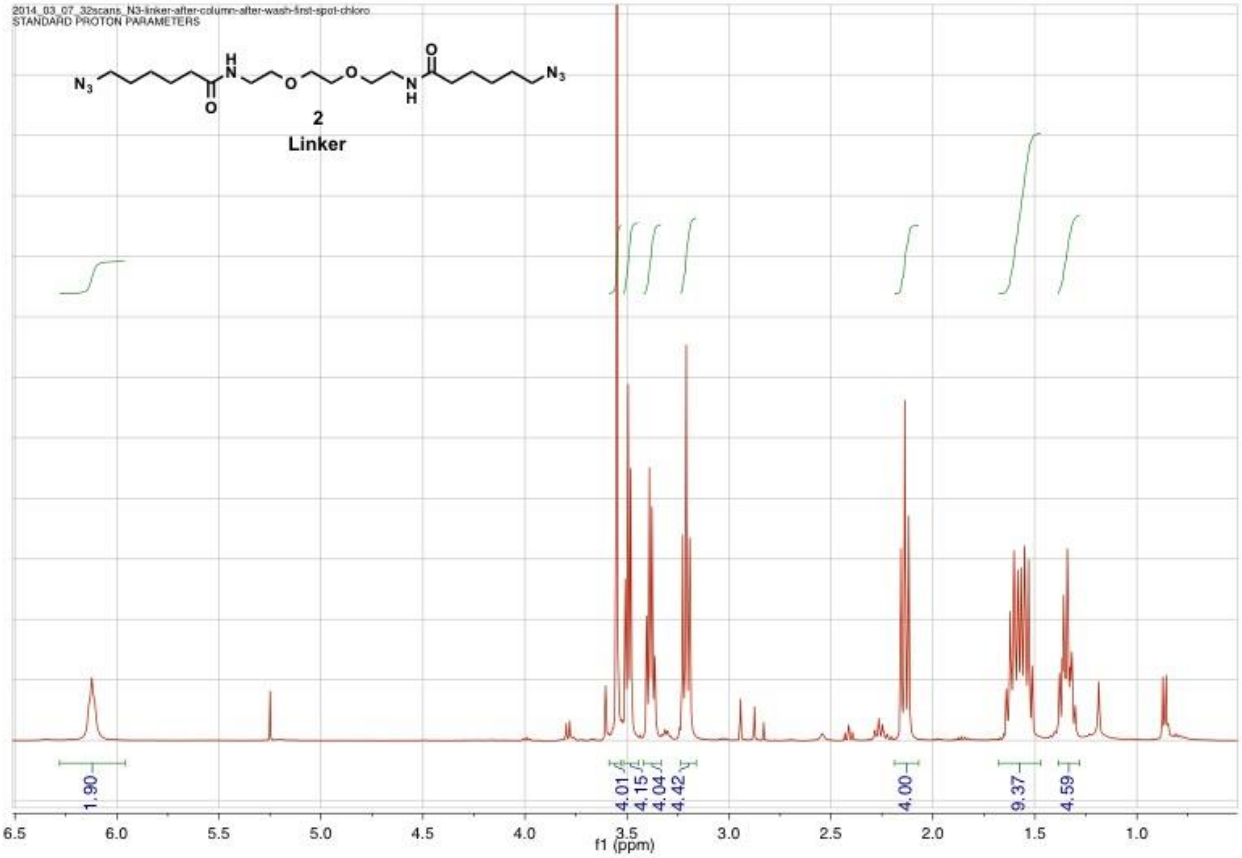

Figure S4: LC-MS of compound **2**

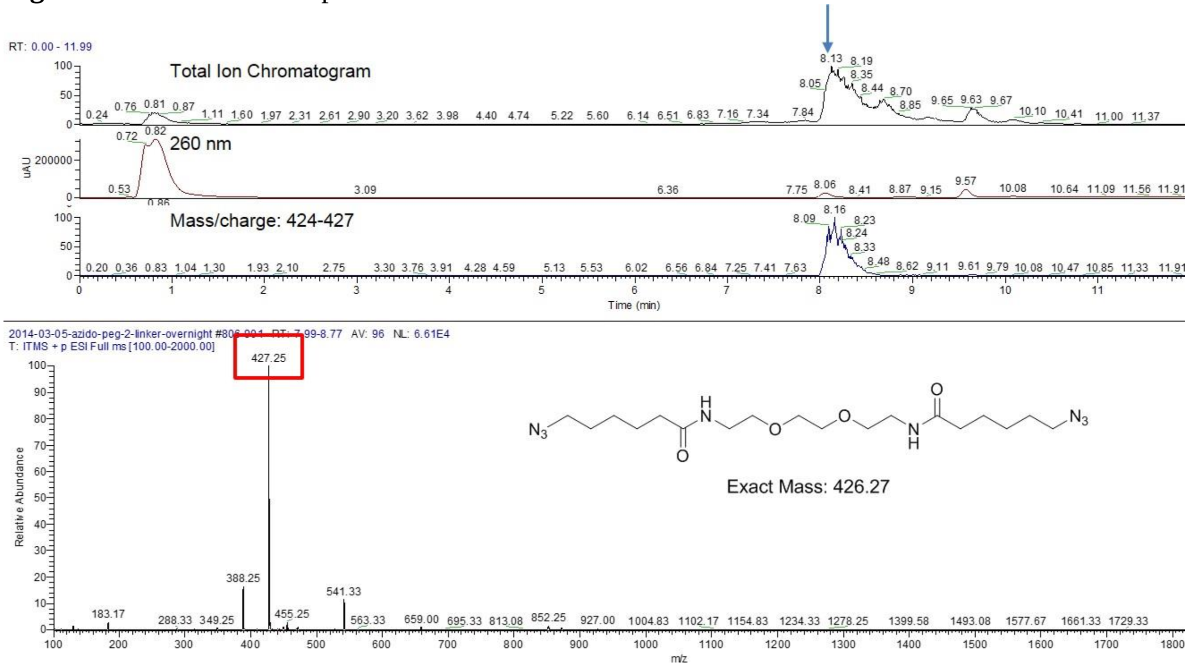

Synthesis of 6-alkyne-F-araNAD$^+$. Synthesis for F-araNMN and 6-alkyne-AMP were done as previously reported and were coupled to make **6-alkyne-F-araNAD$^+$**.(1, 2) First, 6-alkyne-AMP (4.8 mg, 0.012 mmol, 1.0 eq), F-araNMN (4.2 mg, 0.012 mmol, 1.0 eq), and magnesium chloride hexahydrate (58.6 mg, 0.288 mmol, 24 eq) were dissolved in water (3 mL) in a round-bottom flask. Then, the water was evaporated using high vacuum. Next, 0.925 mL MOPS buffer (1.5 M, pH 7.4) was added to dissolve the salt. The solution was transferred to an Eppendorf tube and EDCI (460 mg, 2.4 mmol, 200 eq) was added. The reaction was agitated on a rotating mixer at 37°C incubator for 3.5 h. Subsequently, the reaction was lyophilized and the resulting powder was washed with acetonitrile (3 x 10 mL washes). Subsequently, the product was dissolved in water and purified using preparative HPLC. Preparative HPLC purification was done using AG1 anion exchange resin followed by a C18 reverse phase column purification. For the AG1 anion exchange resin purification (t_R = 34 min), Buffer A was water and Buffer B was 150 mM trifluoroacetic acid in water. The gradient was 0% – 32% buffer B from 0 to 27 min, then 32% – 64% buffer B from 27 to 32 min followed by 64% – 100% buffer B from 32 to 42 min. For the C18 prep HPLC purification (t_R = 27.5 min), the gradient was 0% acetonitrile with trifluoroacetic acid 0-10 min, then 0 - 60% acetonitrile with trifluoroacetic acid from 10 – 70 min. The product was lyophilized and obtained as a solid (0.47 mg, 5.4% yield). LC-MS (ESI) calcd. for $C_{24}H_{29}FN_7O_{13}P_2^+$ (M^+) 704.13, obsd. 704.17.

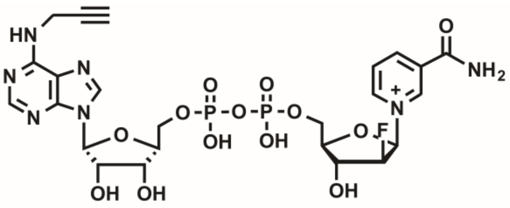

6-alkyne-F-araNAD$^+$

Figure S5: LC-MS of 6-alkyne-F-araNAD$^+$

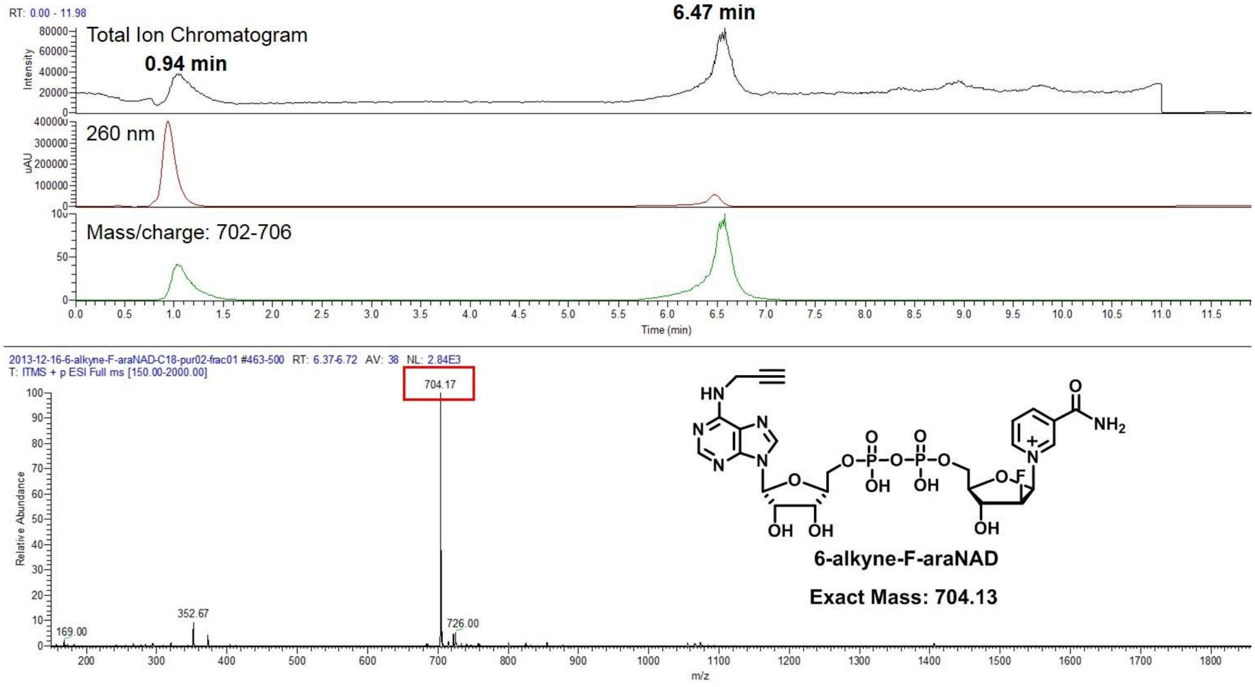

Synthesis of dF-araNAD$^+$ (3). **6-alkyne-F-araNAD$^+$** (2.5 mL of a 0.208 mM aqueous solution, 0.52 µmol, 1.75 eq.), **2** (14.9 µL of a 20 mM DMF solution, 0.30 µmol, 1.0 eq.), Tris[(1-benzyl-1*H*-1,2,3-triazol-4-yl)methyl]amine (the ligand, 17.8 µL of an 100 mM DMF solution, 1.78 µmol, 6.0 eq.), copper sulphate (17.8 µL of a 100 mM aqueous solution, 1.78 µmol, 6.0 eq.), sodium ascorbate (28.5 µL of a 100 mM aqueous solution, 2.85 µmol, 9.6 eq.) were combined in that order in a round-bottom flask fitted with a stir bar. The reaction was stirred at room temperature overnight. Product formation was confirmed using LC-MS (260 nm). The product was purified by preparative HPLC (t_R = 38.5 min) with a gradient of 0% solvent B 0-10 min, 0% - 60% solvent B 10 – 70 min. The product was obtained as a solid (0.154 mg, 28% yield). LC-MS (ESI) calcd. for $C_{66}H_{92}F_2N_{22}O_{30}P_4^{2+}$ (M^+) 1834.53 and [(M^+)/2] 917.26, obsd. 917.67.

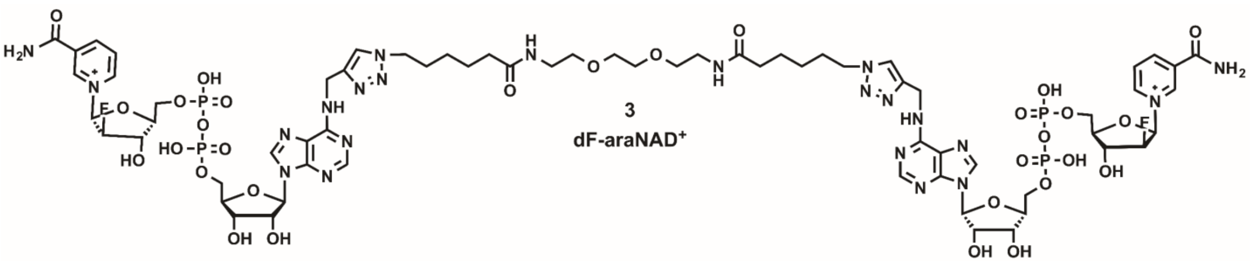

Figure S6: LC-MS of dF-araNAD$^+$

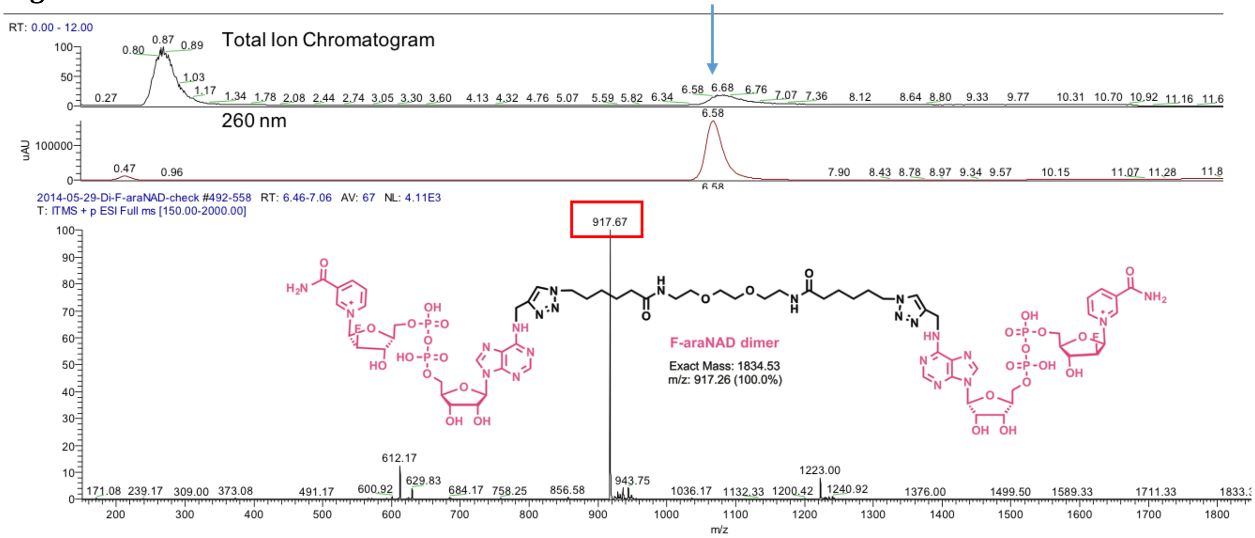

Figure S7: Uncropped western blot images from Figure 2

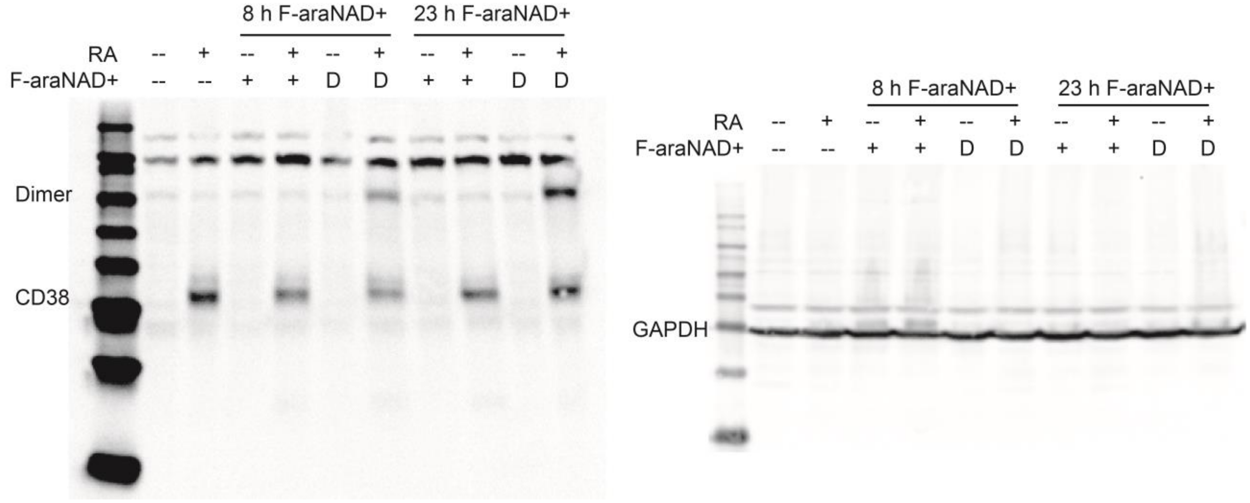

Figure S8: Uncropped western blot images from Figure 4. GAPDH here is also used in Figure 6 because the same representative sets of cellular lysate were used.

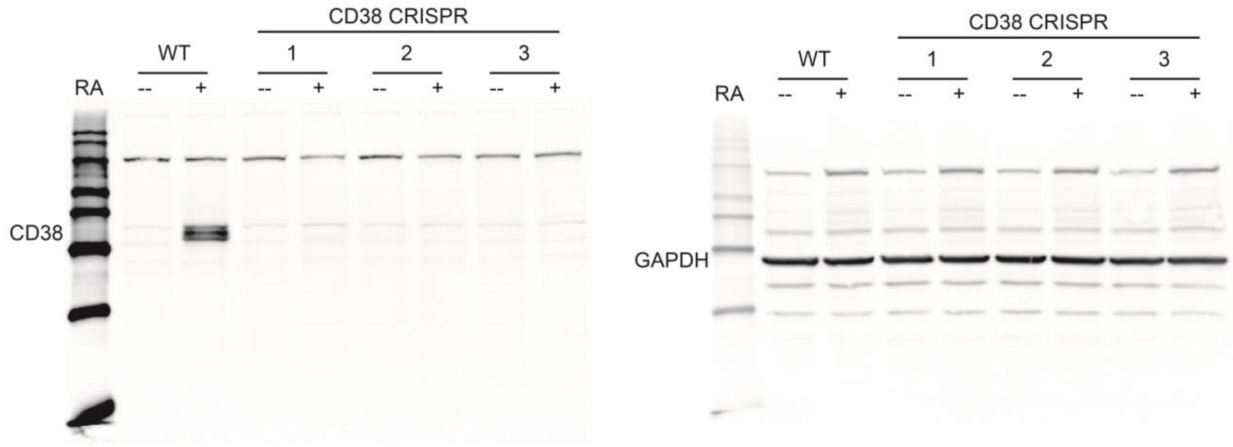

Figure S9: Uncropped western blot images from Figure 6

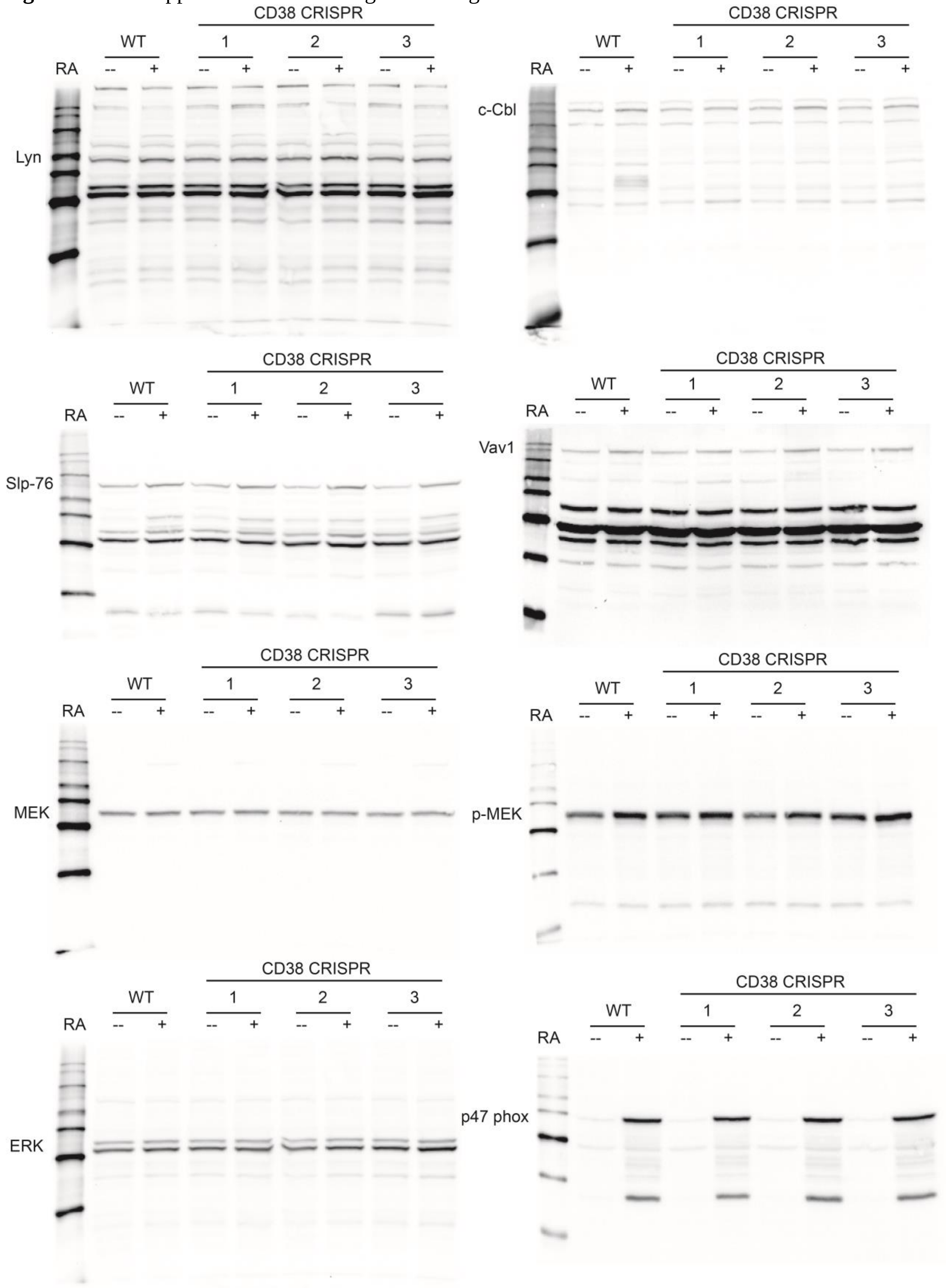

Figure S10: Raw NGD^+ catalysis curves (values from Fluoromax 3). Values at 300 nm excitation/410 nm emission were used when determining RA-induced change in CD38 enzymatic activity.

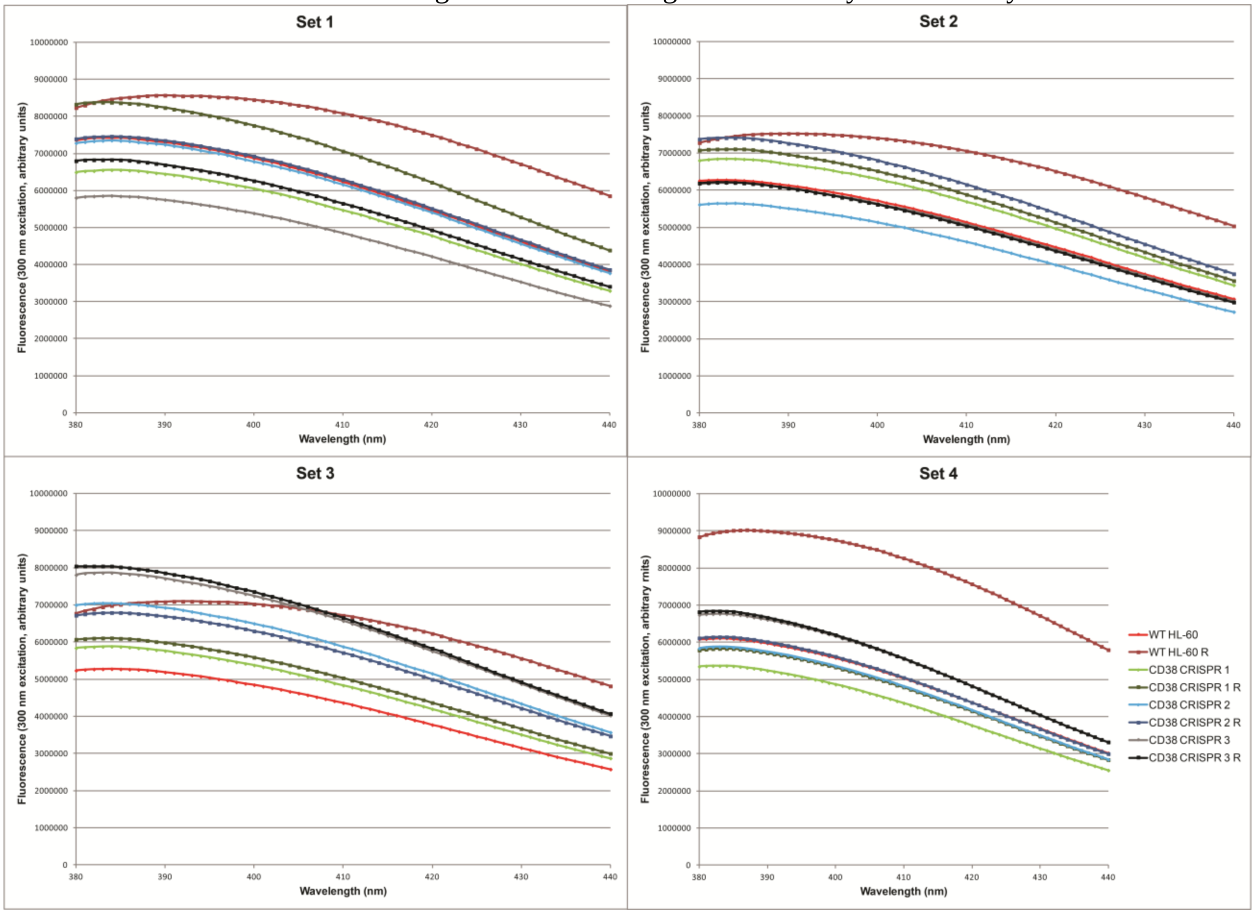

References

1. Shrimp, J. H. *et al.* Revealing CD38 cellular localization using a cell permeable, mechanism-based fluorescent small-molecule probe. *J. Am. Chem. Soc*. **136,** 5656-5663 (2014).
2. Jiang, H. *et al*. Mechanism-based small molecule probes for labeling CD38 on live cells. *J. Am. Chem. Soc.* **131,** 1658–1659 (2009).
